# Robust and Accurate Vision-Based Pose Estimation Algorithm Based on Four Coplanar Feature Points

**DOI:** 10.3390/s16122173

**Published:** 2016-12-17

**Authors:** Zimiao Zhang, Shihai Zhang, Qiu Li

**Affiliations:** Tianjin Key Laboratory of High Speed Cutting & Precision Machining, Tianjin University of Technology and Education, Tianjin 300222, China; zshky77@163.com (S.Z.); liqiu@tute.edu.cn (Q.L.)

**Keywords:** pose estimation, four coplanar points, analytical and iterative, linear constraints, the coordinate system of object motion

## Abstract

Vision-based pose estimation is an important application of machine vision. Currently, analytical and iterative methods are used to solve the object pose. The analytical solutions generally take less computation time. However, the analytical solutions are extremely susceptible to noise. The iterative solutions minimize the distance error between feature points based on 2D image pixel coordinates. However, the non-linear optimization needs a good initial estimate of the true solution, otherwise they are more time consuming than analytical solutions. Moreover, the image processing error grows rapidly with measurement range increase. This leads to pose estimation errors. All the reasons mentioned above will cause accuracy to decrease. To solve this problem, a novel pose estimation method based on four coplanar points is proposed. Firstly, the coordinates of feature points are determined according to the linear constraints formed by the four points. The initial coordinates of feature points acquired through the linear method are then optimized through an iterative method. Finally, the coordinate system of object motion is established and a method is introduced to solve the object pose. The growing image processing error causes pose estimation errors the measurement range increases. Through the coordinate system, the pose estimation errors could be decreased. The proposed method is compared with two other existing methods through experiments. Experimental results demonstrate that the proposed method works efficiently and stably.

## 1. Introduction

With the development of modern industrial technology, quickly and accurately determining the position and orientation between objects is becoming more and more important. This process is called pose estimation. Vision-based pose measurement technology, also known as perspective-n-point (PNP) problem, is to determine the position and orientation of a camera and a target with n feature points on the condition of knowing their world coordinates and 2D image pixel coordinates. It has the advantages of non-contact and high efficiency. It can be widely applied in robotics [1], autonomous aerial refueling [2], electro-optic aiming systems [3,4], virtual reality [5], etc.

The existing approaches to solve object poses can be divided into two categories: analytical algorithms and iterative algorithms. Analytical algorithms apply linear methods to obtain algebraic solutions: Hu et al. [6] uses four points to achieve a linear solution. Lepetit et al. [7] proposed a non-iterative solution named EPnP algorithm. Tang et al. [8] presented a linear algorithm on the condition of five points. Ansar et al. [9] presented a general framework to directly recover the rotation and translation. Duan et al. [10] introduced a new affine invariant of trapezium to realize pose estimation and plane measurement.

As for iterative algorithms, pose estimation is formulated as a nonlinear problem with the constraints, and then it is solved using nonlinear optimization algorithms, most typically, Levenberg-Marquardt method. DeMenthon et al. [11,12,13] proposed POSIT algorithm. It gets the initial value of the solution using a scaled orthographic model to approximate the perspective projection model. Zhang et al. [14,15] proposed a two-stage iterative algorithm. The iterative algorithm is divided into a depth recovery stage and an absolute orientation stage. Peng et al. [16] achieved the object pose non-linearly on the basis of five points using a least squares approach. Liu et al. [17] gets the object pose based on the corresponding geometrical constraints formed by four non-coplanar points. Zhang et al. [18] proposed an efficient solution for vision-based pose determination of a parallel manipulator. Fun et al. [19] proposed a robust and high accurate pose estimation method to solve the PNP problem in real time.

The analytical solutions generally take less computation time. However, they are sensitive to observation noise and usually obtain lower accuracy. As for iterative solutions, the pose estimation is translated into a nonlinear least squares problem through the distance constraints. Then the distance error between feature points is minimized based on 2D image pixel coordinates. However, the iterative solutions also have drawbacks: (1) non-linear optimization needs a good initial estimate of the true solution, (2) they are more time consuming than analytical solutions. 

During the pose estimation process, the image processing errors emerge mainly because the perspective projection point of the feature marker center and the perspective projection image center of feature markers do not coincide. The current papers focus on the reduction of image processing errors through better extraction of the image center [20,21,22]. However, those methods are not powerful enough to eliminate the inconsistency between the perspective projection point of the feature marker center and the center of the corresponding perspective projection image, especially when the measurement range increases. 

Based on the discussion above, in this paper a robust and accurate pose estimation method based on four coplanar points is proposed: In the first step, by utilizing the linear constraints formed by points, the coordinates of points in the camera coordinate system are solved analytically. The results obtained in the first step are then set as the initial values of an iterative solving process to ensure the accuracy and convergence rate of non-linear algorithm. The Levenberg-Marquardt optimization method is utilized to refine the initial values. In the second step, the coordinate system of object motion is established and the object pose is finally solved. The growing image processing error causes greater pose estimation errors with an increasing measurement range. Through the coordinate system, the pose estimation errors could be decreased.

The rest of the paper is organized as follows: Section 2 and Section 3 propose a robust and accurate pose estimation method. Section 4 provides some experiments to examine the method. Section 5 gives the conclusion.

## 2. The Solving of Feature Point Coordinates in the Camera Coordinate System

**Figure 1:**
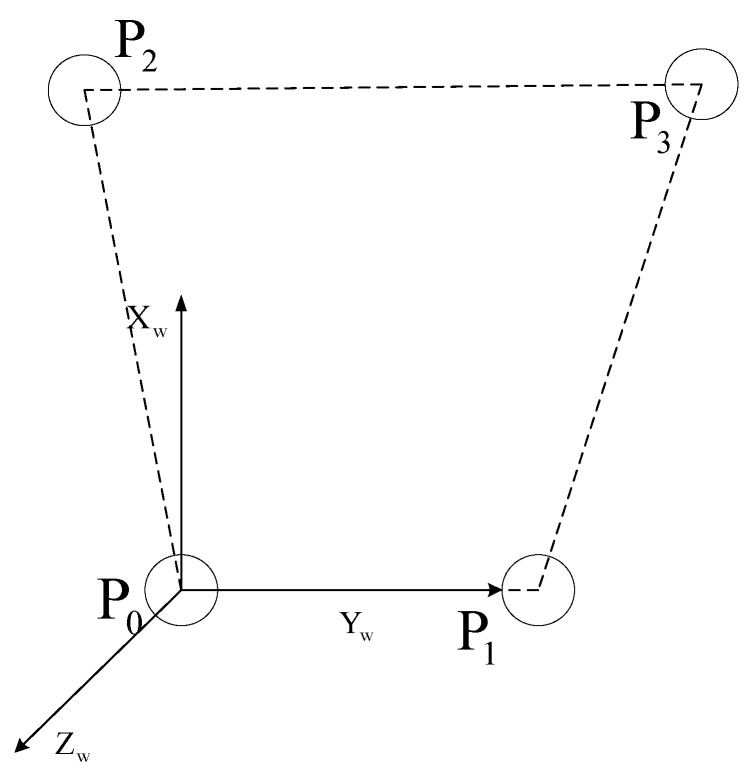
The measurement target with four points.

The target pattern with four coplanar points is designed for pose estimation as shown in Figure 1. P_0_, P_1_, P_2_ and P_3_ form a trapezium. P_0_P_1_ is parallel to P_2_P_3_. 

P_0_ is set as the origin of coordinate system. The connecting line of P_0_ and P_1_ is *Y* axis. $\vec{P_{0}P_{1}}\times \vec{P_{1}P_{3}}$ is *Z* axis. Then *X* axis is $\vec{Z}\times \vec{Y}$. In this way, the world coordinate system (target coordinate system) is constructed.

To achieve the solution of target position, the coordinates of points in the camera coordinate system need to be solved first. $o_{c}-x_{c}y_{c}z_{c}$ is the camera coordinate system. $o_{w}-x_{w}y_{w}z_{w}$ is the world coordinate system. The coordinates of each point in the camera coordinate system is $P_{ci}={\left(x_{ci},y_{ci},z_{ci}\right)}^{T}$. The coordinates of each point in the world coordinate system are $P_{wi}={\left(x_{wi},y_{wi},z_{wi}\right)}^{T}$. $P_{wi}$ is known before solving the pose. The corresponding ideal image coordinates are $I_{ui}={\left(x_{ui},y_{ui},1\right)}^{T}\left(i=0,1,2,3\right)$. The relationship of $P_{ci}$ and $I_{ui}$ could be described as

(1) $$\begin{array}{l} z_{ci}I_{ui}=\left[\begin{array}{ccc} s_{x}f/d_{x} & 0 & u_{0}\\ 0 & f/d_{y} & v_{0}\\ 0 & 0 & 1 \end{array}\right]P_{ci}=KP_{ci}\\ P_{ci}=\lambda_{i}K^{-1}I_{ui}(\lambda_{i}=z_{ci}) \end{array}$$

where (*u*_0_, *v*_0_) is the center pixel of the computer image, *s*_x_ is the uncertainty image factor, *d*_x_ and *d*_y_ are center to center distances between pixels in the row and column directions respectively, *f* is the focal length, and $\lambda_{i}$ is the projection depth of *P_i_*. 

The coordinates of points in the camera coordinate system could be obtained by solving $\lambda_{i}$. Since P_0_P_1_ is parallel to P_2_P_3_, two linear constraints are introduced. Then $\lambda_{i}$ is solved (The solving process is in the Appendix).

(2) $$\begin{array}{l} \lambda_{0}=\sigma_{0}\lambda_{1},\lambda_{2}=\sigma_{2}\lambda_{1}/\varepsilon ,\lambda_{3}=\sigma_{3}\lambda_{1}/\varepsilon \\ \varepsilon =\left\|P_{w1}-P_{w0}\right\|/\left\|P_{w3}-P_{w2}\right\|\\ \left\|P_{w3}-P_{w2}\right\|=\left\|P_{c3}-P_{c2}\right\|=\lambda_{1}\left\|\sigma_{3}K^{-1}I_{u3}/\varepsilon -\sigma_{2}K^{-1}I_{u2}/\varepsilon \right\|\\ \lambda_{1}=\left\|P_{w1}-P_{w0}\right\|/\left\|\sigma_{3}K^{-1}I_{u3}-\sigma_{2}K^{-1}I_{u2}\right\| \end{array}$$

The coordinates of points in the camera coordinates system is obtained by $\lambda_{i}$. So, the vector from the optical center *O*_c_ to each point could be calculated through Equation (3).

(3) $$\begin{array}{l} \vec{O_{c}P_{ci}}=\sigma_{i}K^{-1}I_{ui}\left\|P_{w1}-P_{w0}\right\|/\left\|\sigma_{3}K^{-1}I_{u3}-\sigma_{2}K^{-1}I_{u2}\right\|(i=0,1)\\ \vec{O_{c}P_{ci}}=\sigma_{i}K^{-1}I_{ui}\left\|P_{w3}-P_{w2}\right\|/\left\|\sigma_{3}K^{-1}I_{u3}-\sigma_{2}K^{-1}I_{u2}\right\|(i=2,3) \end{array}$$

The length between the feature points could be calculated through Equation (4).

(4) $$D_{i,\;j}^{2}={\left\|\vec{O_{c}P_{ci}}\right\|}^{2}+{\left\|\vec{O_{c}P_{cj}}\right\|}^{2}-2\vec{O_{c}P_{ci}}\cdot \vec{O_{c}P_{cj}}(i,j=0,1,2,3)$$

In order to maintain the planarity of the four points, the coplanar constraint (CO) expressed as Equation (5) should also be considered.

(5) $$\mathrm{CO}=(\vec{P_{c0}P_{c1}}\times \vec{P_{c2}P_{c3}})\cdot (\vec{P_{c0}P_{c2}}\times \vec{P_{c1}P_{c3}})$$

Through Equation (6), the Levenberg-Marquardt optimization method is then used to solve $\lambda_{i}$. The value of $\lambda_{i}$ obtained in Equation (2) is used as the initial value to ensure the accuracy and convergence speed of the algorithm.

(6) $$\begin{array}{l} D_{i,\;j}^{2}-\left\|P_{wi}P_{wj}\right\|=0\\ CO=0 \end{array}$$

## 3. The Solving of Object Pose

**Figure 2:**
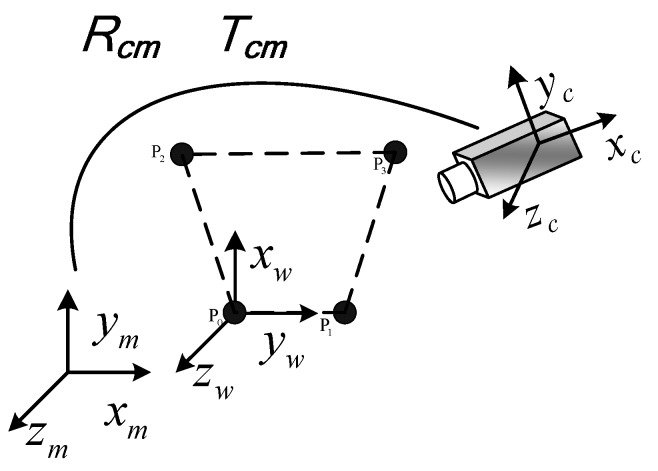
The rotation and translation coordinate system.

The feature points coordinates in the camera coordinate system $P_{c0}$, $P_{c1}$, $P_{c2}$, $P_{c3}$ are calculated through $\lambda_{i}$. The following step is to solve the object pose. As shown in Figure 2, $o_{m}-x_{m}y_{m}z_{m}$ is the rotation and translation coordinate system of target. The three free degrees in rotation are yaw (*y*_m_ axis and α angle), pitch (*x*_m_ axis and β angle), and roll (*z*_m_ axis and γ angle).

$o_{m}-x_{m}y_{m}z_{m}$ is established according to the following steps: (1) The target rotates around *y*_m_ axis. The images of the target are captured in the locations of different rotation angles. N_1_ groups of spatial coordinates $P_{c0}$ can be obtained. With the least squares method, the N_1_ groups of spatial coordinates $P_{c0}$ are used to fit a plane: *Ax* + *By* + *Cz* + *D* = 0. The vector of the *y*_m_ axis is,

(7) $$v={\left[\begin{array}{ccc} r_{4} & r_{5} & r_{6} \end{array}\right]}^{T}={\left[\begin{array}{ccc} \frac{A}{\sqrt{A^{2}+B^{2}+C^{2}}} & \frac{B}{\sqrt{A^{2}+B^{2}+C^{2}}} & \frac{C}{\sqrt{A^{2}+B^{2}+C^{2}}} \end{array}\right]}^{T}$$

(2) The target rotates around *x*_m_ axis. The images of target are captured in the locations of different rotation angles. N_2_ groups of spatial coordinates $P_{c0}$ can be obtained. With the least squares method, the N_2_ groups of spatial coordinates $P_{c0}$ are used to fit a plane: *Ex* + *Fy* + *Gz* + *H* = 0. The vector of the *x*_m_ axis is,

(8) $$u={\left[\begin{array}{ccc} r_{1} & r_{2} & r_{3} \end{array}\right]}^{T}={\left[\begin{array}{ccc} \frac{E}{\sqrt{E^{2}+F^{2}+G^{2}}} & \frac{F}{\sqrt{E^{2}+F^{2}+G^{2}}} & \frac{G}{\sqrt{E^{2}+F^{2}+G^{2}}} \end{array}\right]}^{T}$$

(3) The point sets $P_{c0}$ obtained in steps (1) and (2) share one rotation center. A sphere-fitting is adopted to describe the center. According to the following sphere-fitting equation, the sphere center could be calculated. The sphere center is the rotation center (the origin of $o_{m}-x_{m}y_{m}z_{m}$).

(9) $$\begin{array}{l} \sum_{1}^{N_{1}}{\left(P_{c0x}-x_{om}\right)}^{2}+{\left(P_{c0y}-y_{om}\right)}^{2}+{\left(P_{c0z}-z_{om}\right)}^{2}-r^{2}=0\\ \sum_{1}^{N_{2}}{\left(P_{c0x}-x_{om}\right)}^{2}+{\left(P_{c0y}-y_{om}\right)}^{2}+{\left(P_{c0z}-z_{om}\right)}^{2}-r^{2}=0 \end{array}$$

(*x*_om_, *y*_om_, *z*_om_) is the sphere center and r is the sphere radius.

(4) The vector of the *z*_m_ axis is $w=u\times v={\left[\begin{array}{ccc} r_{7} & r_{8} & r_{9} \end{array}\right]}^{T}$. The vector of the *y*_m_ axis is then adjusted by $v=w\times u={\left[\begin{array}{ccc} r_{4} & r_{5} & r_{6} \end{array}\right]}^{T}$.

As $o_{m}-x_{m}y_{m}z_{m}$ is established, the rotation and translation matrix from $o_{c}-x_{c}y_{c}z_{c}$ to $o_{m}-x_{m}y_{m}z_{m}$ is obtained. The coordinates of feature points in $o_{m}-x_{m}y_{m}z_{m}$ are shown below.

(10) $$\begin{array}{l} R_{cm}=\begin{array}{cc} \left[\begin{array}{ccc} u & v & w \end{array}\right]=\left[\begin{array}{ccc} r_{1} & r_{4} & r_{7}\\ r_{2} & r_{5} & r_{8}\\ r_{3} & r_{6} & r_{9} \end{array}\right] & \;T_{cm}={\left[\begin{array}{ccc} x_{om} & y_{om} & z_{om} \end{array}\right]}^{T} \end{array}\\ P_{mi}=R_{cm}P_{ci}+T_{cm} \end{array}$$

The coordinates of feature points in $o_{m}-x_{m}y_{m}z_{m}$ are represented with $P_{mi}$.

The positioning accuracy of the feature points during image processing have a direct impact on the pose estimation accuracy. The automatic identification of circular markers is more convenient. There are many available algorithms for the center positioning of circular markers. So in this paper, the center point of the circular markers is selected as the feature point. The nature of imaging is the perspective projection. It has the characteristic that the object is big when near and small when far. This causes the perspective projection point of the circular marker’s center and the center of the corresponding perspective projection image (usually an ellipse) to not coincide, especially when the measurement range is greater [23]. This inconsistency causes image processing errors. Then image processing errors emerge, which may result in the pose estimation errors (The ideal image coordinates $I_{ui}={\left(x_{ui},y_{ui},1\right)}^{T}$ in Equation (1) are obtained through these image processing steps). In order to improve measurement accuracy, the following method is introduced.

**Figure 3:**
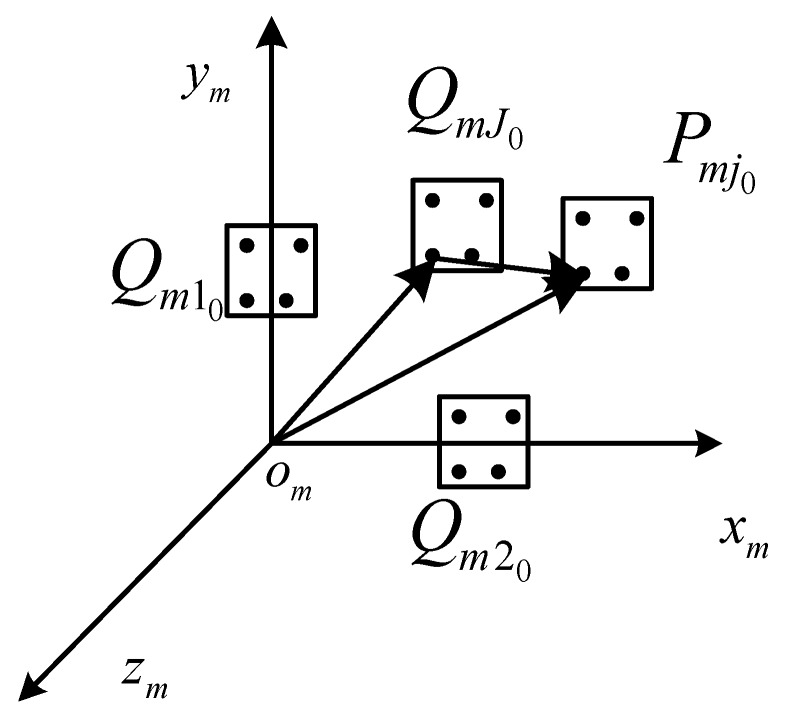
The solving of the object pose.

The point sets $P_{c0}$ obtained in Section 2 represent the known typical position of a target in the moving space. It means N typical positions of a target. The pose of a target in the typical position is known. According to Equation (10), N $P_{m0}$ are obtained. Then the coordinates of $P_{0}$ in $o_{m}-x_{m}y_{m}z_{m}$ in the typical position j are represented with $Q_{mj_{0}}$. $P_{mk_{i}}$ represents the coordinates of feature points in $o_{m}-x_{m}y_{m}z_{m}$ in the location k. The nearest $Q_{mj_{0}}$ to $P_{mk_{0}}$ is $Q_{mJ_{0}}$ as shown in Figure 3. $P_{mk_{i}}$ is calculated as

(11) $$\vec{O_{m}P_{mk_{i}}}=\vec{O_{m}Q_{mJ_{i}}}+\vec{Q_{mJ_{i}}P_{mk_{i}}}\;(i=0,1,2,3)$$

Each pose vector in the moving space corresponds to three angles. They are α (yaw angle), β (pitch angle), and γ (roll angle). They could be calculated as shown in Equation (12).

(12) $$\begin{array}{l} \alpha_{O_{m}P_{mk_{i}}}=\alpha_{O_{m}Q_{mJ_{i}}}+\alpha_{Q_{mJ_{i}}P_{mk_{i}}}\\ \beta_{O_{m}P_{mk_{i}}}=\beta_{O_{m}Q_{mJ_{i}}}+\beta_{Q_{mJ_{i}}P_{mk_{i}}}\\ \gamma_{O_{m}P_{mk_{i}}}=\gamma_{O_{m}Q_{mJ_{i}}}+\gamma_{Q_{mJ_{i}}P_{mk_{i}}} \end{array}$$

$\alpha_{O_{m}Q_{mJ_{i}}}$, $\beta_{O_{m}Q_{mJ_{i}}}$ and $\gamma_{O_{m}Q_{mJ_{i}}}$ are known quantities. $\alpha_{Q_{mJ_{i}}P_{mk_{i}}}$, $\beta_{Q_{mJ_{i}}P_{mk_{i}}}$ and $\gamma_{Q_{mJ_{i}}P_{mk_{i}}}$ could be calculated according to Equation (13). In this way, image processing error caused by the inconsistency mentioned above is significantly reduced, especially when the measurement range is greater. The pose measurement accuracy is then improved. The target pose could be represented with $\alpha_{O_{m}Q_{mk_{i}}}$, $\beta_{O_{m}Q_{mk_{i}}}$, and $\gamma_{O_{m}Q_{mk_{i}}}$.

(13) $$\begin{array}{l} R_{Q}=\left[\begin{array}{cc} \begin{array}{cc} g_{1} & g_{2} \end{array} & g_{3} \end{array}\right]\\ g_{2}=\vec{Q_{mJ0}Q_{mJ1}}/\left|\vec{Q_{mJ0}Q_{mJ1}}\right|\\ l_{1}=\vec{Q_{mJ1}Q_{mJ3}}/\left|\vec{Q_{mJ1}Q_{mJ3}}\right|\\ g_{3}=g_{2}\times l_{1}\\ g_{1}=g_{3}\times g_{2}\\ R_{P}=\left[\begin{array}{cc} \begin{array}{cc} g_{4} & g_{5} \end{array} & g_{6} \end{array}\right]\\ g_{5}=\vec{P_{mk0}P_{mk1}}/\left|\vec{P_{mk0}P_{mk1}}\right|\\ l_{2}=\vec{P_{mk1}P_{mk3}}/\left|\vec{P_{mk1}P_{mk3}}\right|\\ g_{6}=g_{5}\times l_{2}\\ g_{4}=g_{6}\times g_{5}\\ R_{QP}=R_{P}^{-1}R_{Q}\\ R_{QP}=\left[\begin{array}{ccc} \sin\alpha \cos\gamma & \cos\alpha \sin\gamma & -\sin\alpha \\ \sin\beta \sin\alpha \cos\gamma -\cos\beta \sin\gamma & \sin\beta \sin\alpha \sin\gamma +\cos\beta \cos\gamma & \sin\beta \cos\alpha \\ \cos\beta \sin\alpha \cos\gamma +\sin\beta \sin\gamma & \cos\beta \sin\alpha S\gamma -\sin\beta \cos\gamma & \cos\beta \cos\alpha \end{array}\right] \end{array}$$

## 4. Experiment Results

**Figure 4:**
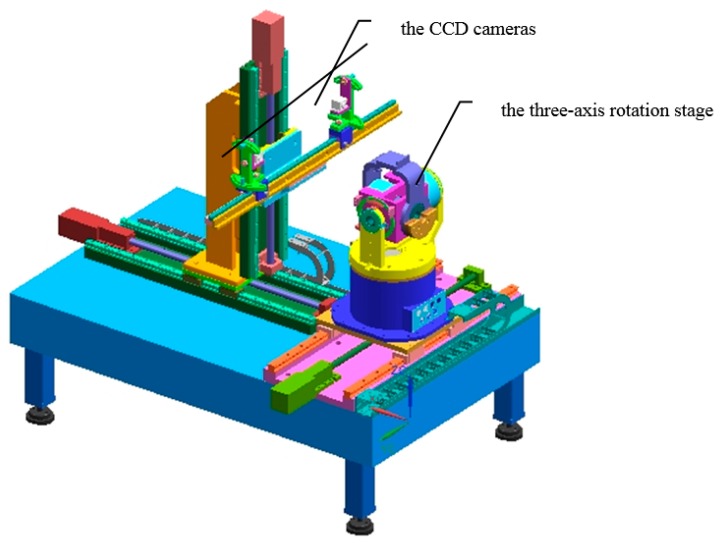
The measurement system.

The experimental system that consists of a target, a three-axis rotation stage, and two CCD cameras is shown in Figure 4. The rotation range of stage in yaw axis, pitch axis, roll axis are ±160°, ±90°, ±60° respectively.

Camera used in this paper is Teli CSB4000F-20 (Teli, Tokyo, Japan) with the resolution 2008 (h) × 2044 (v), pixel size 0.006 × 0.006 mm^2^. The lens is Pentax (Tokyo, Japan) 25 mm. The positioning accuracy of rotation stage is less than 20″. The calibration results of camera intrinsic parameters are shown in Table 1 [24]. *k*_1_, *k*_2_ are the radial distortion coefficients. *p*_1_, *p*_2_ are the tangential distortion coefficients.

The images are captured with a single CCD camera in different locations. The feature point coordinates in the camera coordinate system are obtained in the current location. Then the rotation and translation coordinate system of target $o_{m}-x_{m}y_{m}z_{m}$ is established. $\alpha_{O_{m}Q_{mk_{i}}}$, $\beta_{O_{m}Q_{mk_{i}}}$ and $\gamma_{O_{m}Q_{mk_{i}}}$ which could be used to represent the target pose are solved.

The target pose measurement experiment includes three parts: computer simulation experiments, real image experiments for accuracy, and real image comparative experiments with other methods.

### 4.1. Computer Simulation Experiments

**Figure 5:**
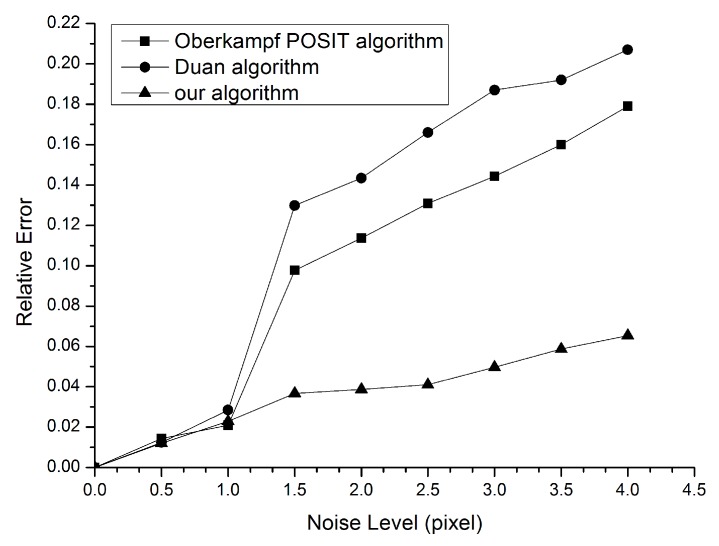
The simulation experiment results.

To validate accuracy and noise immunity of the algorithm in this paper, the algorithm proposed is compared with the Oberkampf POSIT algorithm [11,12,13] and the Duan algorithm [10] during the computer simulation experiment process. In this process, the pinhole imaging model of a camera is simulated, thus the points are transformed with perspective projection and the simulated image coordinates of points are acquired. Gaussian noise from 0 pixels to 4 pixels are added to the point’s coordinates of images. The relative errors of estimated poses using the proposed algorithm, Duan algorithm, and Oberkampf POSIT algorithm are shown in Figure 5. 

It can be seen that the errors are reduced after optimization and they increase with the noise level. It is noticeable that the method proposed produces better results than the other two methods, especially when the noise level is greater than one pixel. In addition, it is noted that the errors keep almost the same level under lower noise disturbance. This phenomenon can be explained as follows: owing to the fact that the Duan algorithm does not have an iterative solving process, this leads to a relatively high error. The Oberkampf POSIT method takes no account of both the coplanarity constraint and initial value of iteration process. With the noise level increasing, the factor of error changes from initial value of the iteration process to coplanarity. This results in a higher error than the proposed algorithm.

### 4.2. The Measurement Experiments for Accuracy

**Figure 6:**
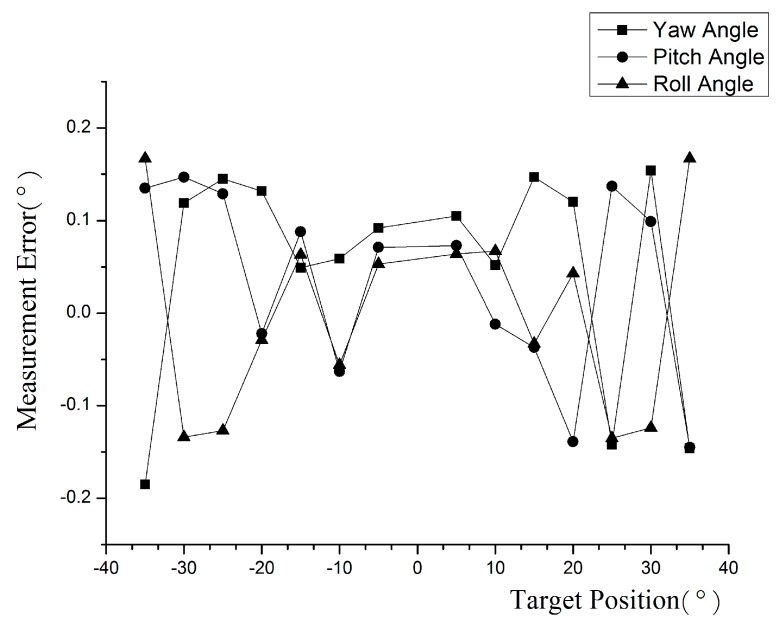
The measurement error of yaw, pitch, and roll angle.

The measurement range of yaw angle, pitch angle, and roll angle is set to −35°~35°. The measurement errors are shown in Figure 6. The measurement error is the absolute difference of the estimated angle to the true angle.

### 4.3. The Comparative Experiments for Accuracy

**Figure 7:**
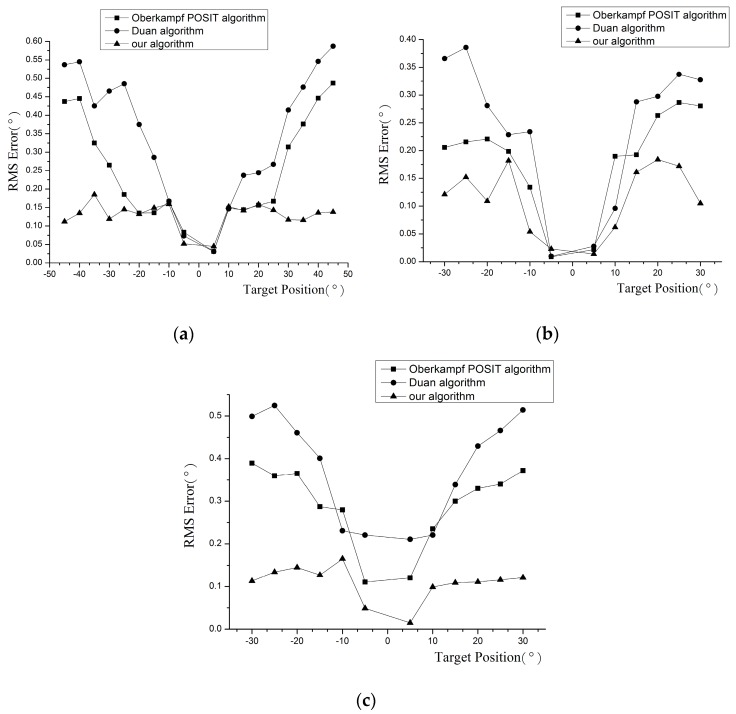
The RMS error of rotation angles: (**a**) Yaw angle; (**b**) Pitch angle; (**c**) Roll angle.

The algorithm proposed in this paper, Duan algorithm, and Oberkampf POSIT algorithm are used to calculate target poses. The root mean square (RMS) errors are displayed in Figure 7. Each point in the figure is the RMS error over multiple measurements. As to the Duan algorithm, when the rotation angle is greater, the RMS error of our method is less than that of Duan algorithm. The experiment results demonstrate that the analytical pose measurement process is sensitive to the observation noise. This error could be reduced by the iterative process in our method. At the same time, in our algorithm a good initial value is provided at the beginning of iteration to ensure pose estimation accuracy. As to the Oberkampf POSIT algorithm, experiment results demonstrate that the image processing error mentioned in Section 3 exists in the pose measurement process. Through the method in Section 3, the error could be eliminated successfully, especially when the measurement range is greater. 

By comparing the results of our method and those of the Oberkampf POSIT and Duan algorithms, it is obvious that the measurement accuracy of our method is higher than those in the whole moving space.

**Figure 8:**
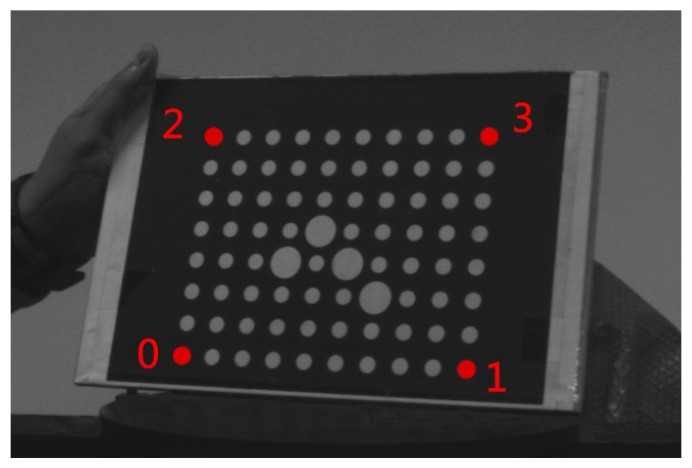
The image of P_0_, P_1_, P_2_, and P_3_ on the calibration board.

**Figure 9:**
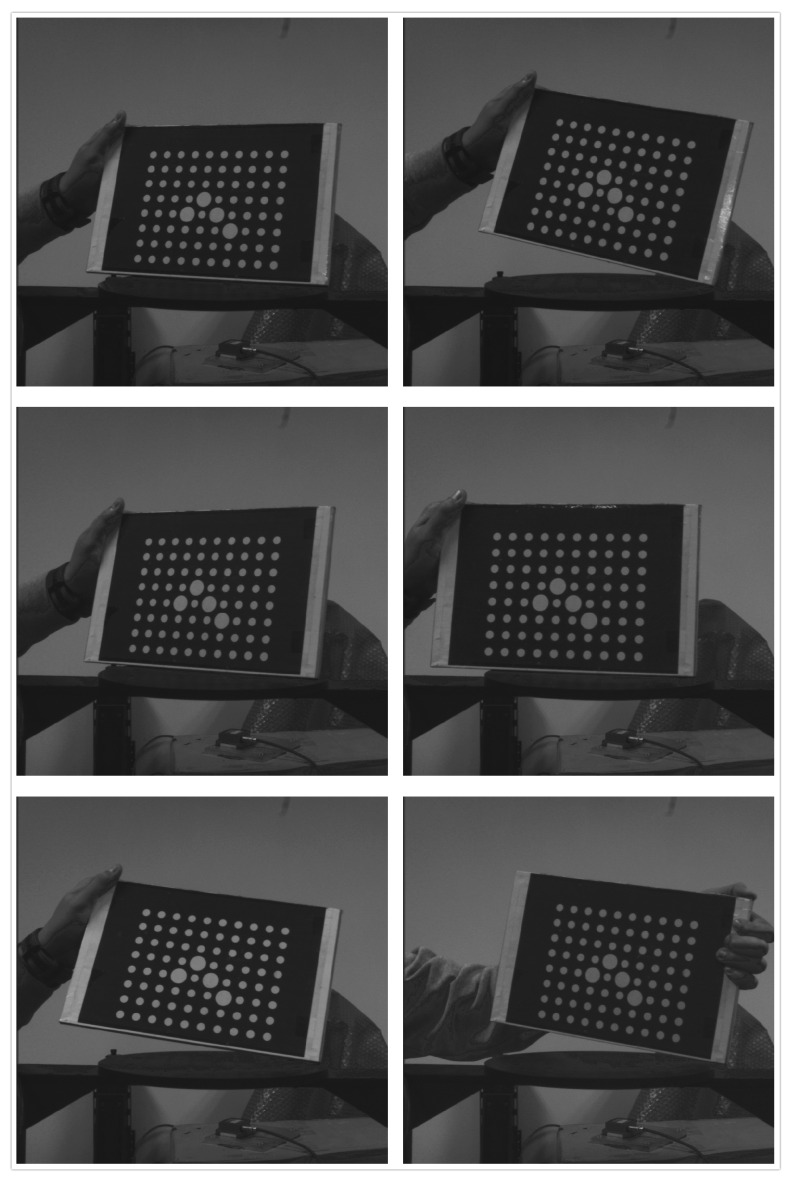
The image sets of the calibration board.

As to the real applications, we first take images for the calibration board of the camera, as shown in Figure 8. The four corner circular markers (marked in red) of the calibration board are used to form a trapezium. The center points of the four circular markers are extracted from the images. Then the lengths of the four sides of the trapezium are calculated with the algorithm proposed in this paper, Duan algorithm, and Oberkampf POSIT algorithm. The measurement errors are shown in Table 2. Figure 9 shows the image sets of the calibration board. D_01_ is the distance between P_0_ and P_1_. D_02_ is the distance between P_0_ and P_2_. D_23_ is the distance between P_2_ and P_3_. D_13_ is the distance between P_1_ and P_3_. The measurement error is the absolute difference of the estimated length to the true length. It can be seen from Table 2 that the results of our algorithm are more close to the real value. The measurement error of our method is less than 0.2 mm. The algorithm proposed in this paper is more effective. 

**Figure 10:**
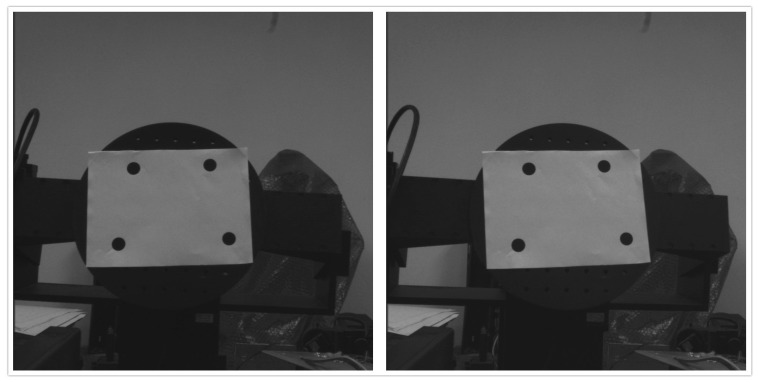
The image of the rotation stage with feature points.

Then we test the proposed algorithm by experimentally detecting the pose information of a rotation stage in yaw angle, pitch angle, and roll angle. Figure 10 shows the image sets of the rotation stage with feature points. Through the target on the rotation stage as shown in Figure 10, its pose is calculated using the algorithm proposed in this paper, Duan algorithm, and Oberkampf POSIT algorithm. The measurement errors are shown in Table 3. The measurement error is the absolute difference of the estimated angle to the true angle. It can be seen from Table 3 that the results of our algorithm are more close to the real value. The measurement error of our method is less than 0.2°. Using the same information extracted from the images, the proposed algorithm has an advantage to identify the pose of the rotation stage.

**Figure 11:**
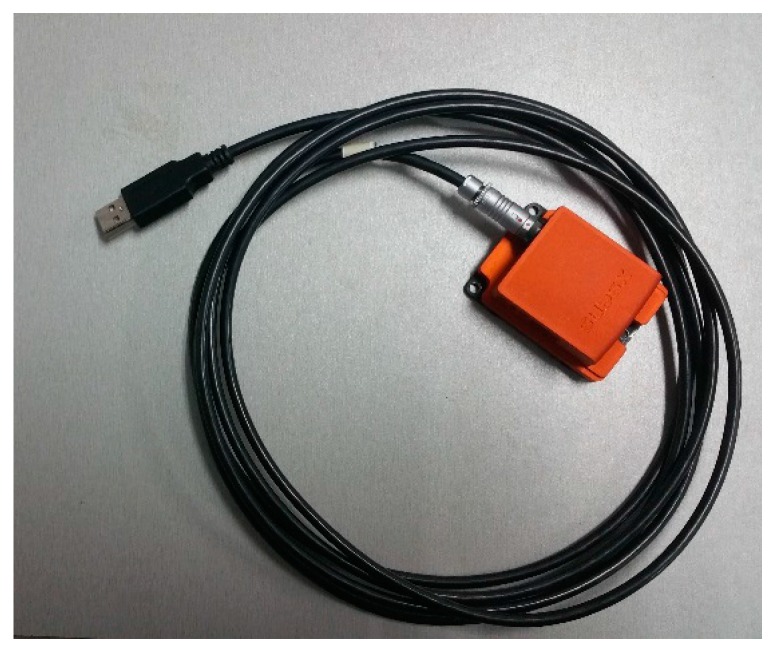
The motion capture system based on inertial technology.

**Figure 12:**
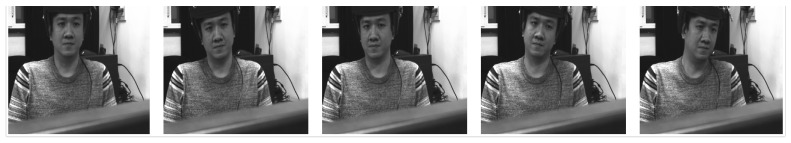
The image sets of head pose.

Finally, we estimated the head pose. The pose measurement system based on inertial technology is mounted on a helmet to calculate the head pose as shown in Figure 11. The results are taken as the real values. The corresponding images of the head are captured and shown in Figure 12. The vertices of the trapezium are the outer corners of the two eyes and the mouth. The image points of the four vertices on each image are located in a manual way. The head pose is calculated using the algorithm proposed in this paper, Duan algorithm, and Oberkampf POSIT algorithm. The first image (Frame 0) in Figure 12 is set as the initial position (zero position). The angle between the current positions (Frame 1, Frame 2, Frame 3, and Frame 4) and the initial position (Frame 0) are calculated. The measurement errors are shown in Table 4. The measurement error is the absolute difference of the estimated angle to the real angle.

It can be seen from Table 4 that the results of our algorithm are closer to the real value. The rough localization of the control points (the trapezium) in the image lead to the promotion of the measurement errors. However, our method could decrease these kinds of measurement errors compared with the other two methods. The errors of yaw, pitch, and roll are not independent. The errors of yaw, pitch, and roll influence each other for they constitute the rotation matrix together. When the head rotated in all the axes, as the errors of yaw, pitch, and roll influence each other, measurement errors are promoted.

The distance between object and camera is limited by the field of view. The corresponding experiment results (RMS errors) are shown in Table 5. When the camera is in different locations, the pose of the target is calculated. In Position 1 and Position 2, the camera is near to the target and the target is out of the field of view of the camera. In Position 5 and Position 6, the camera is far from the target and the target is out of the field of view of the camera. In Position 3 and Position 4, the target is in of the field of view of the camera. It can be seen as follows: if the object is out of the range of the field of view, the measurement accuracy is lower. If the object is in the range of the field of view, the measurement accuracy is higher and little influenced by the distance between object and camera. So in order to obtain a higher accuracy, it is needed to ensure that the object is in the range of the field of view of the camera.

## 5. Conclusions

A robust and accurate vision-based pose estimation algorithm based on four coplanar feature points is presented in this paper. By combining the advantages of the analytical methods and the iterative methods, the iterative process is given a preferable initial value acquired independently by an analytical method. At the same time, the anti-noise ability is strengthened and the result is more stable. The pose estimation errors depend on the feature extraction accuracy. When the measurement range is greater, the image processing errors become greater. In the algorithm proposed, the coordinate system of object motion is established to solve the object pose. In this way, the image processing error which may result in the pose estimation errors could be reduced. 

## Figures and Tables

**Table 1:** Calibration results.

Parameters	*f_x_*	*f_y_*	*c_x_*	*c_y_*	*k*_1_	*k*_2_	*p*_1_	*p*_2_
Camera 1	4341.501	4341.918	1034.667	1033.926	−0.361	0.140	−0.00024	0.00008
Camera 2	4373.530	4373.659	1000.997	1020.977	−0.373	0.342	0.00033	−0.00040

**Table 2:** The measurement errors of the calibration board.

	Frame 1	Frame 2	Frame 3	Frame 4	Frame 5	Frame 6
**D_01_ (mm)**						
Duan	−0.455	0.456	−0.352	0.568	−0.672	−0.433
Oberk	0.338	−0.209	0.189	−0.209	0.335	−0.326
Our	−0.047	0.148	−0.078	−0.041	0.039	−0.086
**D_02_ (mm)**						
Duan	0.532	−0.622	−0.535	0.659	−0.553	0.436
Oberk	0.248	−0.295	0.306	−0.331	0.359	−0.389
Our	0.040	−0.138	0.025	0.114	0.052	0.088
**D_23_ (mm)**						
Duan	0.555	−0.636	−0.418	−0.659	−0.559	0.736
Oberk	−0.323	0.203	−0.353	0.268	−0.177	0.384
Our	0.063	0.055	0.156	−0.125	−0.029	−0.139
**D_13_ (mm)**						
Duan	−0.708	−0.654	−0.555	0.715	−0.453	−0.359
Oberk	0.448	−0.407	−0.348	0.207	−0.248	0.307
Our	−0.027	−0.072	0.076	−0.165	−0.089	−0.134

**Table 3:** The measurement errors of the rotation stage with feature points.

	Position				
Yaw	5°	10°	15°	20°	25°
Duan	−0.132	0.252	0.245	0.485	−0.532
Oberk	−0.056	−0.154	−0.147	0.284	−0.334
Our	0.032	−0.057	0.045	−0.085	−0.132
Pitch (error)	5°	10°	15°	20°	25°
Duan	−0.127	−0.196	−0.201	0.386	−0.477
Oberk	0.046	0.177	−0.186	0.295	0.402
Our	0.027	0.043	−0.019	−0.102	−0.137
Roll	5°	10°	15°	20°	25°
Duan	0.129	−0.267	0.245	−0.399	−0.489
Oberk	0.036	−0.200	−0.196	0.247	−0.374
Our	−0.026	−0.052	0.049	0.093	0.140

**Table 4:** The measurement errors of head pose.

	Frame 1	Frame 2	Frame 3	Frame 4
**Duan (°)**				
Yaw	−0.845	−0.831	−0.776	0.766
Pitch	0.476	−0.552	0.544	0.422
Roll	−0.577	0.430	0.402	−0.427
**Oberk (°)**				
Yaw	−0.560	−0.620	−0.688	−0.644
Pitch	0.410	−0.374	−0.397	0.294
Roll	−0.355	−0.288	0.362	−0.403
**Our (°)**				
Yaw	−0.345	0.344	0.286	−0.374
Pitch	−0.177	−0.186	−0.211	0.204
Roll	−0.222	−0.199	0.175	0.232

**Table 5:** The RMS error of cameras in different locations.

	Position 1	Position 2	Position 3	Position 4	Position 5	Position 6
Yaw (°)	0.411	0.384	0.192	0.189	0.477	0.512
Pitch (°)	0.400	0.372	0.186	0.177	0.444	0.504
Roll (°)	0.394	0.346	0.174	0.185	0.500	0.523

## Appendix A. Proof of the Solving of $\lambda_{i}$

ε is the distance ratio of P_1_P_0_ and P_3_P_2_.

(A1) $$\begin{array}{l} \varepsilon =\left\|P_{w1}-P_{w0}\right\|/\left\|P_{w3}-P_{w2}\right\|\\ P_{w1}=\varepsilon (P_{w3}-P_{w2})+P_{w0}=\left[P_{w0},P_{w2},P_{w3}\right]{\left[1,-\varepsilon ,\varepsilon \right]}^{T} \end{array}$$

The transformation matrix from the world coordinates system to the camera coordinates system is **R** and **T**. $P_{ci}$ could be expressed as $P_{ci}=R\cdot P_{wi}+T$, and then $P_{c1}$ could be represented with $P_{c0}$, $P_{c2}$, $P_{c3}$. The first linear constraint is shown in Equation (A2).

$$\begin{array}{l} P_{c1}=R\cdot P_{c4}+T\\ =R\cdot (\varepsilon (P_{c3}-P_{c2})+P_{c0})+T\\ =R\cdot (\varepsilon (P_{c3}-P_{c2}+T-T)+P_{c0})+T\\ =\varepsilon (R\cdot P_{c3}-R\cdot P_{c2}+T-T)+R\cdot P_{c0}+T\\ =\varepsilon (P_{c3}-P_{c2})+P_{c0} \end{array}$$

(A2) $$P_{c1}=\varepsilon (P_{c3}-P_{c2})+P_{c0}=\left[P_{c0},P_{c2},P_{c3}\right]{\left[1,-\varepsilon ,\varepsilon \right]}^{T}$$

Given $P_{c}=[P_{c0},P_{c2},P_{c3}]$, according to Equations (1) and (A2), Equation (A3) is deduced.

(A3) $$P_{c}=[P_{c0},P_{c2},P_{c3}]=K^{-1}[I_{u0},I_{u2},I_{u3}]diag[\lambda_{0},\lambda_{2},\lambda_{3}]$$

The both sides of Equation (A3) are multiplied with $P_{c1}$. Given $[\sigma_{0},\sigma_{2},\sigma_{3}]={\left[I_{u0},-I_{u2},I_{u3}\right]}^{-1}I_{u1}$, finally Equation (A4) is obtained which represents the second linear constraint.

(A4) $$P_{c}^{-1}P_{c1}=diag(\lambda_{1}/\lambda_{0},\lambda_{1}/\lambda_{2},\lambda_{1}/\lambda_{3}){\left[\sigma_{0},-\sigma_{2},\sigma_{3}\right]}^{T}$$

$P_{c}^{-1}P_{c1}$ could also be expressed as Equation (A5) according to Equation (A2).

(A5) $$P_{c}^{-1}P_{c1}={\left[1,-\varepsilon ,\varepsilon \right]}^{T}$$

According to both Equations (A4) and (A5), $\lambda_{i}$ is solved as shown in Equation (2) in the paper.
